# Supplementary material for: Towards establishing a fungal economics spectrum in soil saprobic fungi
Source: Nat Commun. 2024 Apr 18;15:3321. doi: 10.1038/s41467-024-47705-7 (PMC11026409; doi:10.1038/s41467-024-47705-7)
Supplement: Supplementary file 1 — Supplementary Information [file 41467_2024_47705_MOESM1_ESM.pdf]

Supplementary Information S1

Paper: **Towards establishing a fungal economics spectrum in soil saprobic fungi**

Journal: Nature Communications

Authors: Camenzind T, Aguilar-Trigueros C, Hempel S, Lehmann A, Bielcik M, Andrade-Linares DR, Bergmann J, dela Cruz J, Gawronski J, Golubeva P, Haslwimmer H, Lartey L, Leifheit E, Maaß S, Marhan S, Pinek L, Powell JR, Roy J, Veresoglou SD, Wang D, Wulf A, Zheng W, Rillig MC

Table S1 Description of all fungal functional and fundamental niche traits included in this study, displaying the name of individual studies (Table S2), an overview of applied methodologies and the ecological interpretation and relevance of each trait.

Fungal trait	Study ID[1]	Average value / PC axis extracted	Method[2]	Trait relevance
**extension**	DALx, Alx, AL20, WZ20, TC20, TC22, CATx, TCx, JAdC22, EL23	Average of standardized values derived from all data	Mycelial extension rate of fungi grown on agar plates - new mycelial area formed within defined periods of time within the linear growth phase, assessed under optimal growth conditions (no resource limitation)	Mycelial exploration potential; fast vs. slow growth type
**density**	WZ20, TC20, TC22, CATx, TCx, EL23	Average of standardized values derived from all data	Mycelial density of fungi grown on agar plates - mycelial biomass per area observed at the end of experiments, measured under optimal growth conditions (no resource limitation)	Mycelial exploitation potential; fast vs. slow growth type
**$biomass_{st}$**	WZ20, TC20, TC22, CATx, TCx, EL23, PG20, DW20	Average of standardized values derived from all data	Mycelial biomass of fungi formed on growth media - total fungal biomass measured at the end of experiments, assessed under optimal growth conditions (no resource limitation)	Fungal growth yield under standard conditions

**biomass$_{opt}$**	TC20, TCx, CATx	Average of standardized values derived from all data	Fungal biomass values measured at species-specific optima within environmental gradients (C:N supply, temperature, osmotic potential)	Fungal growth yield potential (at optimal growth conditions)
**biomass$_{complC}$**	EL23	Average of standardized values derived from all data	Fungal biomass values obtained on media with more complex C sources (xylan, cellulose and mixed leaf litter)	Fungal growth yield in the absence of simple sugars
**CUE**	TCxc		Fungal carbon-use efficiency (CUE) assessed on fungi grown on 10% PDA - respired C / (respired C + biomass C)	Carbon-use efficiency
**hyphal_diam**	AL19		Hyphal diameter [ym] - measured at the last internode before the tip; fungi were grown on concavity slides with 10% PDA for microscopic examination	Hyphal architecture
**melanin**	TCxd		Melanin content in fungal biomass based on a quantitative colorimetric assay	Melanin content in fungal cell walls is (i) related to environmental stress tolerance and (ii) soil C sequestration [1]
**water_cont**	WZ20		Mycelial water content [%] analyzed with fungi grown on PDA overlain by a cellophane layer; derived from analyses of dry vs. wet fungal biomass	Hyphal chemical composition; potentially related to water demands
**hydrop**	WZ20		Mycelial hydrophobicity measured by the alcohol percentage test on mycelia grown on PDA overlain with a cellophane layer	Related to production of hydrophobins; relevant for hyphal growth patterns in soil
**DNAc**	SMx		DNA concentration in fungal mycelia [µg g^{-1} fungus] analyzed on fungi grown on malt extract broth	Indicator of mycelial activity
**PLFAc**	SMx		Total PLFA (phospholipid fatty acids) content [nmol g^{-1} fungus] analyzed on fungi grown on malt extract broth	Hyphal chemical composition; PLFA are partly related to C storage mechanisms
**stoich_C_X$_{PC1}$**	TC21		PC1 of C:X (nutrient) values measured on defined media with C:N 20 - positive	Hyphal chemical composition; element demands

			values correlate with high C:X values (43% var expl)	
**stoich_N_X$_{PC1}$**	TC21		(Inverted) PC1 of N:X values measured on defined media with C:N 20 - positive values correlate with high N:X values (57% var expl)	Hyphal chemical composition; element demands
**stoich_N_X$_{PC2}$**	TC21		(Inverted) PC2 of N:X values measured on defined media with C:N 20 - positive values correlate with high N:K values (low K content; 25% var expl)	Hyphal chemical composition; element demands
**C_cont**	TC21		C content [%] assessed after growth on defined media with C:N 20	Hyphal chemical composition; fungal C demands; potentially also related to storage
**enz_la**	WZ20		Enzymatic activity of laccase measured on mycelial pieces by a microplate photometric method; after growth on PDA overlain with cellophane	Hyphal ability to produce laccase, a complex enzyme for lignin degradation
**enz_leu**	WZ20		Enzymatic activity of leucine aminopeptidase measured on mycelial pieces by a microplate photometric method; after growth on PDA overlain with cellophane	Indication of fungal N demands
**enz_cel**	WZ20		Enzymatic activity of cellobiohydrolase measured on mycelial pieces by a microplate photometric method; after growth on PDA overlain with cellophane	Hyphal ability to produce cellobiohydrolase, a complex enzyme for cellulose degradation
**enz_pho**	WZ20		Enzymatic activity of acid phosphatase measured on mycelial pieces by a microplate photometric method; after growth on PDA overlain with cellophane	Indication of fungal P demands
**complC_use**	EL23		Complex Carbon Use Ability - indicator of the ability of fungal isolates to grow on more complex C sources; measured as the weighted average of relative biomass values on media with different C sources: relative biomass on glucose x 1, cellobiose x 2, xylan x 3, cellulose x 4, litter x 5 / sum of relative biomass	Indicator of fungal capacity to use and degrade complex C substrates, unbiased by potentially species-specific enzyme complexes

**enz_C_div**	EL23		C enzyme diversity based on rapid enzymatic essays using the API ZYM^{TM} kit; counts of the number of C enzymes for which fungal activity was observed	Indicator for the presence of more complex C enzymes in fungi
**spore_abund**	TC22	Average of standardized values on 1% and 100% PDA	Spore abundance [spores mg^{-1} fungus] produced by fungi after 6 weeks of growth on 1% and 100% PDA - asexual spores (except for Chaetomium isolates); since most isolates sporulated differently under those conditions (though values strongly correlated, see publication), average values were taken	Fungal sporulation as an indicator of dispersal and colonization potential of new resource patches; fungal investment into spores
**spore_shape**	TC22	Aaverage of standardized values on 1% and 100% PDA	Fungal spore shape [length / width] - asexual spores (except for Chaetomium isolates); since values varied slightly depending on growth media (though values strongly correlated, see publication), average values were taken	Architectural trait, also related to dispersal agent and width
**spore_size**	TC22	Average of standardized values on 1% and 100% PDA	Fungal spore size [µm] - asexual spores (except for *Chaetomium* isolates); since values varied slightly depending on growth media (though values strongly correlated, see publication), average values were taken	Spore size relates fungal investment into spores, and potentially spore establishment success
**spore_RRx**	TC22		Relative increase in spore production under nutrient scarcity - log response ratio spore abundance 1% / 100% PDA	Fungal strategy to invest into spore production under resource limitations → escape mechanism via spores
**stoich_flex**	TC21		Stoichiometric C:N flexibility of mycelia [homeostatic coefficient $1/H_{CN}$]; indicator of non-homeostatic behaviour in C:N ratios of fungi measured along a gradient of C:N 5-200	Ability to flexibly adjust to N limitations; related to mycelial flexibility/recycling
**recycling**	TCxb		Fungal internal resource recycling ability; measured as the relative new area formed by fungal mycelia supported only by internal fungal	Internal resource recycling ability to support hyphal extension

			recycling activity [new area formed / initial area]	
**asegurl**	TC22, EL23, TC20		Ability to Switch to Explorative Growth Under Resource Limitation, based on the observation that most isolates switch to explorative growth (increased mycelial extension) under resource limitations; this trait was quantified as an increase in extension rate under resource limiting conditions (determined based on the decrease in biomass compared to optimum supply); - slope coefficient (RRextension rate ~ RRbiomass change); see Fig. S6	Ability to switch to explorative growth under resource limitation
**WA_explor**	SV18		Higher mycelial extension on WA (resource limited, agar as sole C source) compared to PDA (rich medium, glucose as C source) on split plates - log response ratio extension WA / extension PDA)	Investment into exploration; preference for complex C sources over glucose
**comp_glu**	SS18	PC1 axis extracted from all competition scores (90% var. expl.)	Competitive interaction outcomes of isolate pairs grown on PDA were tested; after 4 weeks of growth the outcomes of each pairwise competition were measured by win/loss/neutral scores, as well as fungal growth compared by intra- vs. interspecific growth patterns	Fungal competitive ability in the presence of simple sugars
**fungic_str**	JAdC22		Fungal stress tolerance to fungicide addition (isopyrazam, 1 mg L^{-1}); log response ratio of mycelial extension rate with / without fungicide	Stress tolerance to fungicide

**cu_str**	PG20		Fungal stress tolerance to copper additions (1 mM), grown in PDB - log response ratio biomass with copper / without	Stress tolerance to copper
**drought_str**	TCx		Fungal stress tolerance to drought - growth response (biomass) to drought induced by PEG additions to PDB (-1.91 MPa) - log response ratio biomass at -1.91 MPa / biomass at -0.49 MPa (control, PDB)	Stress tolerance to drought
**stress_tol$_{av}$**	JAdC22, PG20, TCx	Average of standardized values of stress responses	Average of standardized values of stress tolerances (fungicide, copper, drought); positive values indicate higher stress tolerance	Stress tolerance
**stress_tol$_{PC1}$**	JAdC22, PG20, TCx	PC1 axis of PCA of all stress responses (43% var. expl.)	PC1 axis of PCA of all stress responses (fungicide, copper, drought); positive values correlate to stress tolerance; more strongly related to drought and fungicide than copper stress	Stress tolerance
**soil_aggreg**	AL20		Soil aggregate formation potential [%], soil aggregates >1mm size formed de novo by fungal isolates	Soil aggregate formation potential
**leaf_decomp**	EL23		Leaf litter decomposition rate [%] of *Arrhenatherum elatius* litter after 10 weeks of fungal inoculation; measured in litter bags (petri dish design)	Leaf litter decomposition potential
**wood_decomp**	EL23		Wood litter decomposition rate [%] of *Tilia cordata* wood after 10 weeks of fungal inoculation; measured in litter bags (petri dish design)	Wood litter decomposition potential
**decomp$_{PC1}$**	EL23	PC1 axis of PCA of litter decomposition (78% var. expl.)	PCA based on leaf litter and wood litter decomposition ability values	Average litter decomposition potential

Fundamental niche dimensions

**N_opt**	TC20		Isolate-specific optima in medium C:N supply, defined by maximum biomass growth on defined media; growth curves were modeled based on skew-normal distribution (C:N gradient of 5, 20, 40, 80, 200)	Fundamental N niche optimum
**N_breadth**	TC20		Isolate-specific niche breadth [>25% of maximum growth] in medium C:N supply, defined by skew-normal distribution based on biomass data	Fundamental N niche breadth
**temp_opt**	CATx		Isolate-specific temperature optimum, defined by maximum biomass growth on PDA under different temperature regimes (12, 15, 18, 21, 24, 27, 30, 33°C); growth curves were modeled based on skew-normal distribution	Fundamental temperature niche optimum
**temp_breadth**	CATx		Isolate-specific niche breadth [>25% of maximum growth] in temperature, defined by skew-normal distribution based on biomass data	Fundamental N niche breadth
**water_opt**	TCxa		Isolate-specific optima in water supply, defined by maximum biomass growth on PDB under a water potential gradient (addition of polyethylene glycol (PEG), -0.49, -0.68, -0.87, -1.2, -1.9); growth curves were modeled based on skew-normal distribution	Fundamental N niche optimum
**water_breadth**	TCxa		Isolate-specific niche breadth [>25% of maximum growth] in water supply, defined by skew-normal distribution based on biomass data	Fundamental N niche breadth
**niche matrix**	TC20, CATx, TCxa		Isolate-specific optima in the three-dimensional niche matrix, with the three axes representing gradients of C:N supply, water potential and temperature	Optimal growth in the complete niche space
**niche_cube**	TC20, CATx, TCxa		The complete three-dimensional niche space for individual isolates (cube) was calculated assuming a cuboid niche shape, with side lengths of the respective relative niche breadths (standardized by the respective maximum niche breadth)	Complete niche breadth in whole measured niche space

[1]see Table S2 for details on the individual studies and further method descriptions; studies marked by an x instead of the date represent unpublished datasets

[2]media abbreviations: PDA: potato-dextrose agar, PDB: potato-dextrose broth

Table S2. Published and unpublished studies included in the dataset, with main traits used here marked in bold (further trait were included to make average traits or included in principal component analyses (PCA) to extract PC axes of trait syndromes).

Study	ID	Title/Topic	Traits used here from original study[1]	Additional (unpublished) analyses and methods used for the dataset	Methodological overview[2]
**Published studies**					
**Lehmann *et al.* 2019** [2]	AL19	"Tradeoffs in hyphal traits determine mycelium architecture in saprobic fungi"	Hyphal diameter (**hyphal_diam**)		
**Lehmann *et al.* 2020** [3]	AL20	"Fungal traits important for soil aggregation"	Mycelial extension rate during linear growth phase on potato-dextrose agar (ext_PDA_AL20); soil aggregate formation potential [%] (**soil_aggreg**)		
**Zheng *et al.* 2020** [4] **(more data in related dissertation** [5]**)**	WZ20	"Growth rate trades off with enzymatic investment in soil filamentous fungi"	Mycelial water content [%] (**water_cont**); mycelial hydrophobicity (**hydrop**); enzymatic activity of leucine aminopeptidase (**enz_leu**) and acid phosphatase (**enz_pho**), extension rate, mycelial density and biomass assessed on PDA and PDA overlain with cellophane layer (ext_PDA_WZ20, ext_cPDA_WZ20, den_PDA_WZ20, den_cPDA_WZ20, bio_PDA_WZ20, bio_cPDA_WZ20)	The enzymatic activities of cellobiohydrolase (**enz_cel**) and laccase (**enz_la**) were repeated with further developed methods [6]	

**Camenzind *et al.* 2022** [7]	TC22	"Soil fungi invest into asexual sporulation under resource scarcity, but trait spaces of individual isolates are unique"	Mycelial extension rate, density and biomass on PDA (ext_PDA_TC22, den_PDA_TC22, bio_PDA_TC22); average spore abundance [spores mg-1], spore shape [length / width] and spore size [µm²] after 6 weeks of growth on 1% and 100% PDA (**spore_abund, spore_shape, spore_size**)	Relative change in spore production under nutrient scarcity - log response ratio spore abundance 1% / 100% PDA (**spore_RRx**)	
**Camenzind *et al.* 2020** [6]	TC20	"Trait-based approaches reveal fungal adaptations to nutrient-limiting conditions"	Mycelial extension rate, density and biomass on media with standard C:N ratios of 20 (ext_CN20_TC20, den_CN20_TC20, bio_CN20_TC20); biomass at species-specific optimum C:N medium (bio_optN_TC20); trait variability data in C:N gradient (Fig. S8); **fungal niche along C:N gradient** (C:N 5, 20, 40, 80, 200)	Traits were complemented for the complete set of 31 fungal isolates; calculations of niche optima were optimized in this study	
**Camenzind *et al.* 2021** [8]	TC21	"Soil fungal mycelia have unexpectedly flexible stoichiometric C:N and C:P ratios"	Fungal stoichiometric flexibility in C:N ratios [1/H_{CN}] along a medium C:N gradient of C:N 5 - 200 (**stoich_flex**); C content [%] in mycelia grown at C:N 20 (**C_cont**)	Traits were complemented for the complete set of 31 fungal isolates; measurements of other element contents (P, S, K, Mg) in fungi grown at C:N20 were added (**stoich_C_**X_{PC1}**, stoich_N_**X_{PC1}**, stoich_N_**X_{PC2})	Other element contents were measured in fungi grown on C:N 20 media only. Element contents were determined after aqua regia digestion (1:4 HCl:HNO3) by ICP-OES analyses (Optima 2100 DV, Perkin Elmer, Germany)

**de la Cruz *et al.* 2022** [9]	JAdC22	"Sub-lethal fungicide concentrations both reduce and stimulate the growth rate of non-target soil fungi from a natural grassland"	Mycelial extension rate on PDA (ext_PDA_JAdC22); growth response to fungicide (isopyrazam) additions at concentration of 1 mg L^{-1} medium - log response ratio extension rate with fungicide / without fungicide (**fungic_str**)	
**Leifheit *et al.*, in press** [10]	EL23	"Fungal traits help to understand the decomposition of simple and complex plant litter"	Mycelial extension rate, density and biomass on defined medium with glucose (ext_GLU_EL23, den_GLU_EL23, bio_GLU_EL23); biomass formed on complex C sources (bio_XYL_EL23, bio_CEL_EL23, bio_LIT_EL23; **biomass$_{\mathbf{complc}}$**); index of complex carbon use ability (**complex_C_use**) - weighted average of relative biomass on media with C sources varying in complexity; litter decomposition rate [%] of wood and leaf litter (**leaf_decomp, wood_decomp**) measured in litter bags (petri dish design)	C enzyme diversity based on rapid enzymatic essays with the API ZYM^{TM} kit - the numbers of C enzymes present (**enz_C_div**)
**Golubeva *et al.* 2020** [11]	PG20	"Soil saprobic fungi differ in their response to gradually and abruptly delivered copper"	Fungal biomass in Czapek-Dox broth medium (bio_CDB_PG20); fungal stress response to copper additions (1 mM) - log response ratio biomass with copper / without copper (**cu_str**)	Traits were complemented for the complete set of 31 fungal isolates
**Wang 2020** [12]	DW20	Dissertation "Trade-offs in soil filamentous fungi"	Fungal biomass in potato dextrose broth (bio_PDB_DW20)	
**Soliveres *et al.* 2012** [13]	SS18	"Intransitive competition is common across five major taxonomic groups and is driven by productivity, competitive	Competitive interaction outcomes between pairwise fungal interactions - different scores assessed based on interaction outcome scores as well as different growth patterns on inter- versus intra-specific interaction plates	PC1 axis extracted from a PCA based on different competition scores (**comp_glu**)

		rank and functional traits"	(comp_scoresums_SS18, comp_winperc_SS18, fcomp_radia_SS18, comp_distia_SS18)	
**Veresoglou *et al.* 2018** [14]	SV18	"Fungal decision to exploit or explore depends on growth rate"	Relative mycelial extension on water agar (WA) compared to rich potato-dextrose agar (PDA) on split plates with the concurrent supply of both resources (log response ratio extension WA / extension PDA). High values indicate wide growth on WA, which can be interpreted as explorative growth (**WA_explor**)	
**Camenzind *et al.*, in press** [32]	TCxa	chemical composition of different fungal isolates in PLFA (phospholipid fatty acid analyses) and DNA concentrations	DNA concentration and PLFA content of mycelia (**DNAc, PLFAc**)	DNA concentrations [µg g^{-1} fungus] (PicoGreen quantification) and PLFA contents [nmol g^{-1} fungus] (standard Bligh and Dyer extraction [17]) were analyzed in fungal isolates following 2 weeks of growth on malt extract broth.
**Unpublished datasets**				

Authors involved in the study				
**Andrade-Linares, D**	DALx	Mycelial growth on different agar media	Mycelial growth on PDA (ext_PDA_DALx)	The average colony diameter was measured after 8 days of fungal growth
**Aguilar-Trigueros, CA**	CATx	Fungal responses to a temperature gradient	Mycelial extension rate, density and biomass on PDA at standard growth temperatures (21°C and 24°C; ext_PDA21_CATx, ext_PDA24_CATx, den_PDA21_CATx, den_PDA24_CATx, bio_PDA21_CATx, bio_PDA24_CATx); biomass at isolate-specific optimum temperature (bio_optT_CATx); trait variability data in temperature gradient (Fig. S8b); **fungal niche along temperature gradient** (12, 15, 18, 21, 24, 27, 30, 33°C)	Fungal isolates were grown on PDA overlain with cellophane at different temperature regimes, with individual incubators representing the experimental unit (n=2). Extension rate was measured as the slope of regression during the linear growth phase (before 200 hours). Fungal biomass and density were determined at the end of the experimental period (12 days).
**Camenzind, T, Gawronski, J**	TCx	Fungal responses to a water potential gradient	Mycelial extension rate, density and biomass on PDB (ext_PDB_TCx, den_PDB_TCx, bio_PDB_TCx); biomass at species-specific optimum water potential (bio_optO_TCx); drought stress - log response ratio of biomass at -1.91MPa / biomass at -0.49 MPa (**drought_str**); **fungal niche along water availability gradient** (-1.91, -1.06, -0.87, -0.68, -0.49 MPa)	The water potential was modified by the addition of polyethylene glycol (PEG) to PDB, following values given by [15]. The liquid medium was added to capillary mats, overlain with cellophane [16]. Mycelial extension rate (during the linear growth phase), density and biomass were determined as response variables.

**Camenzind, T, Lartey, L**	TCxb	Internal fungal resource recycling activity	Internal resource recycling ability - relative new mycelial area formed in the absence of external resources (**recycling**)	Mycelia of fungal isolates (pre-grown for 7 days on defined media (C:N 20; see [6]) overlain with cellophane) were rinsed to remove media leftovers. The pure mycelium was transferred to empty petri dishes and only supplied with H_2O solutions (including NaCl (0.8 g L^{-1}) to avoid osmotic stress). In this design, no resources were available except for the own mycelial tissues. The relative mycelial extension after 14 days of growth on empty petri dishes (new area formed / initial area) was assessed as an indicator of internal resource recycling ability

**Camenzind, T**	TCxc	Measurement of fungal carbon-use efficiency (CUE)	Fungal carbon-use efficiency (**CUE**)	CUE of fungi was assessed in fungi grown on 10% PDA (overlain with a cellophane sheet). Agar plates were placed in air-tight sterile 500ml containers, to capture CO_2 produced during the whole growth period [18]. After a growth period of 7 days (14 in case of insufficient biomass production) fungal biomass was quantified, as well as fungal sC contents (analyzed with an Elemental Analyser (EuroEA, HekaTech, Germany). CUE was calculated following the fomula: CUE = respired C / (respired C + biomass C).
**Camenzind, T**	TCxd	Melanin contents	**melanin**	Following a method proposed by [1], melanin contents in fungi were assessed using a quantitative colorimetric assay based on azure A dye. Isolates were grown for 7 days (10 days in case of slow growth) on PDA overlain by cellophane. Freeze-dried fungal material was weighed into eppis (3 mg) and shaken with 600 ml azure A dye solution for 30 minutes. The final absorption of the solution (610 nm) corresponds to melanin contents [1].

**Lehmann, A**	ALx	Measurement of colony extension rate	Colony extension rate [mm] (ext_PDA_Alx)	Following the inoculation of PDA plates with poppy seeds, the radial colony extension rate was assessed within the first 24 hours

[1]abbreviations growth media: PDA (potato-dextrose agar), PDB (potato-dextrose broth)

[2]methodological details are only given for unpublished studies and analyses

Supplementary Note 1: Details on fungal isolates

Soil samples for fungal isolation were taken in May and September/October 2013 - 2014 in a protected grassland site in Northeastern Germany ('Oderhänge Mallnow' close to the town of Lebus, Germany; 52°28`N, 14°29`E) down to a soil depth of 20 cm. A sampling permit (RO7/SOB-0951A to I) was obtained from the federal environmental agency Brandenburg (Landesumweltamt Brandenburg, Außenstelle Frankfurt/Oder, RO 7 - Naturschutz). Soil samples were washed through 250 and 53 µm sieves, and dilutions of the captured soil material plated on Malt Extract Agar, Benomyl Agar, Rose Bengal Agar, Yeast Extract-Peptone-Dextrose Agar and Czapek Dox Agar[19]. To determine fungal identity, Individual marker regions of long sequence reads were compared to respective databases (Unite database[20] and RDP LSU dataset[21]) using the function assignTaxonomy() (dada2[22]) with a bootstrap threshold of 80%. Taxon names were only accepted in case different markers provided the same result (Table S3).

The original collection of the Rillig Lab Core Set (RLCS) includes 31 isolates, with some of them affiliated to the same genus, i.e., *Chaetomium* (3), *Fusarium* (4) and *Mortierella* (5)[3]. Especially the fungi belonging to *Fusarium* and *Mortierella* show strong trait similarity (see Fig. S9). Therefore, to achieve a more balanced phylogenetic design, only three fungal isolates of the same genus were included in the final dataset. Isolates most dissimilar in trait space (determined by location in PCA space, Fig. S9) and phylogeny were kept (Table S3).

For maintenance of fungal cultures, fungal isolates were transferred to new PDA media irregularly (once or twice a year). Over time, cultures were also renewed from original stock cultures (conserved either in 10% glycerol (-80°C), mineral oil (4°C) or 20% skim milk (freeze dried, 4°C); glycerol stocks were most reliable).

Table S3. Details on fungal isolates included in this study (isolates excluded from main analyses are indicated in grey; phylogeny according to Unite database[20])

strain ID	DSMZ accession number	NCBI accession number	phylum	class	order	family	taxon name
RLCS01	DSM100293	KT582076	Mucoromycota	Mucoromycetes	Mucorales	Mucoraceae	*Mucor fragilis*
RLCS02	DSM100407	KT582072	Mortierellomycota	Mortierellomycetes	Mortierellales	Mortierellaceae	*Mortierella sp.3*
RLCS03	DSM100285	KT582067	Mortierellomycota	Mortierellomycetes	Mortierellales	Mortierellaceae	*Mortierella alpina2*
RLCS04	DSM100322	KT582094	Mortierellomycota	Mortierellomycetes	Mortierellales	Mortierellaceae	*Mortierella sp.2*
RLCS05	DSM100403	KT582097	Ascomycota	Sordariomycetes	Hypocreales	Nectriaceae	*Fusarium sp.1*
RLCS06	DSM100400	KT582096	Ascomycota	Sordariomycetes	Sordariales	Chaetomiaceae	*Chaetomium angustispirale1*
RLCS07	DSM100284	KT582088	Ascomycota	Sordariomycetes	Xylariales	Bartaliniaceae	*Truncatella angustata*
RLCS08	DSM100325	KT582087	Ascomycota	Sordariomycetes	Hypocreales	Nectriaceae	*Fusarium sp.3*
RLCS09	DSM100406	KT582071	Basidiomycota	Agaricomycetes	Polyporales	Polyporaceae	*Trametes versicolor*
RLCS10	DSM100286	KT582078	Ascomycota	Dothideomycetes	Pleosporales	Pleosporaceae	*Alternaria sp.*
RLCS11	DSM100289	KT582070	Mortierellomycota	Mortierellomycetes	Mortierellales	Mortierellaceae	*Mortierella alpina1*
RLCS12	DSM100405	KT582079	Ascomycota	Sordariomycetes	Sordariales	Chaetomiaceae	*Chaetomium angustispirale2*
RLCS13	DSM100290	KT582073	Ascomycota	Sordariomycetes	Hypocreales	Nectriaceae	*Fusarium sp.2*
RLCS14	DSM100404	KT582077	Ascomycota	Dothideomycetes	Pleosporales	Didymellaceae	*Nothophoma sp.*

RLCS15	DSM100402	KT582092	Mortierellomycota	Mortierellomycetes	Mortierellales	Mortierellaceae	*Mortierella sp.1*
RLCS16	DSM100408	KT582080	Basidiomycota	Agaricomycetes	Agaricales	Pleurotaceae	*Pleurotus sp.*
RLCS17	DSM100324	KT582089	Basidiomycota	Agaricomycetes	Agaricales	Entolomataceae	*Clitopilus sp.*
RLCS18	DSM100287	KT582068	Ascomycota	Sordariomycetes	Hypocreales	Nectriaceae	*Fusarium gibbosum*
RLCS19	DSM100331	KT582093	Mucoromycota	Umbelopsidomycetes	Umbelopsidales	Umbelopsidaceae	*Umbelopsis isabellina*
RLCS20	DSM100329	KT582081	Ascomycota	Sordariomycetes	Hypocreales	Ophiocordycipitaceae	*Purpureocillium lilacinum*
RLCS21	DSM100327	KT582065	Ascomycota	Dothideomycetes	Pleosporales	Cucurbitariaceae	*Pyrenochaetopsis leptospora*
RLCS22	DSM100401	KT582091	Ascomycota	Dothideomycetes	Pleosporales	Phaeosphaeriaceae	*Paraphoma chrysanthemicola*
RLCS23	DSM101519	KT582090	Ascomycota	Sordariomycetes	Hypocreales	Stachybotryaceae	*Paramyrothecium sp.*
RLCS24	DSM100410	KT582066	Ascomycota	Sordariomycetes	Hypocreales	Clavicipitaceae	*Metarhizium marquandii*
RLCS25	DSM100292	KT582083	Ascomycota	Sordariomycetes	Hypocreales	Bionectriaceae	*Gliomastix sp.*
RLCS26	DSM100330	KT582084	Ascomycota	Leotiomycetes	Helotiales	Helotiaceae	*Tetracladium apiense*
RLCS27	DSM100326	KT582086	Ascomycota	Sordariomycetes	Sordariales	Chaetomiaceae	*Chaetomium subspirilliferum*
RLCS28	DSM100323	KT582085	Ascomycota	Leotiomycetes	Helotiales	NA	*NA*
RLCS29	DSM100288	KT582069	Basidiomycota	Agaricomycetes	Agaricales	Agaricaceae	*Macrolepiota excoriata*
RLCS30	DSM100291	KT582075	Ascomycota	Eurotiomycetes	Chaetothyriales	Herpotrichiellaceae	*Exophiala sp.*
RLCS31	DSM100328	KT582074	Ascomycota	Eurotiomycetes	Chaetothyriales	Cyphellophoraceae	*Cyphellophora sp.*

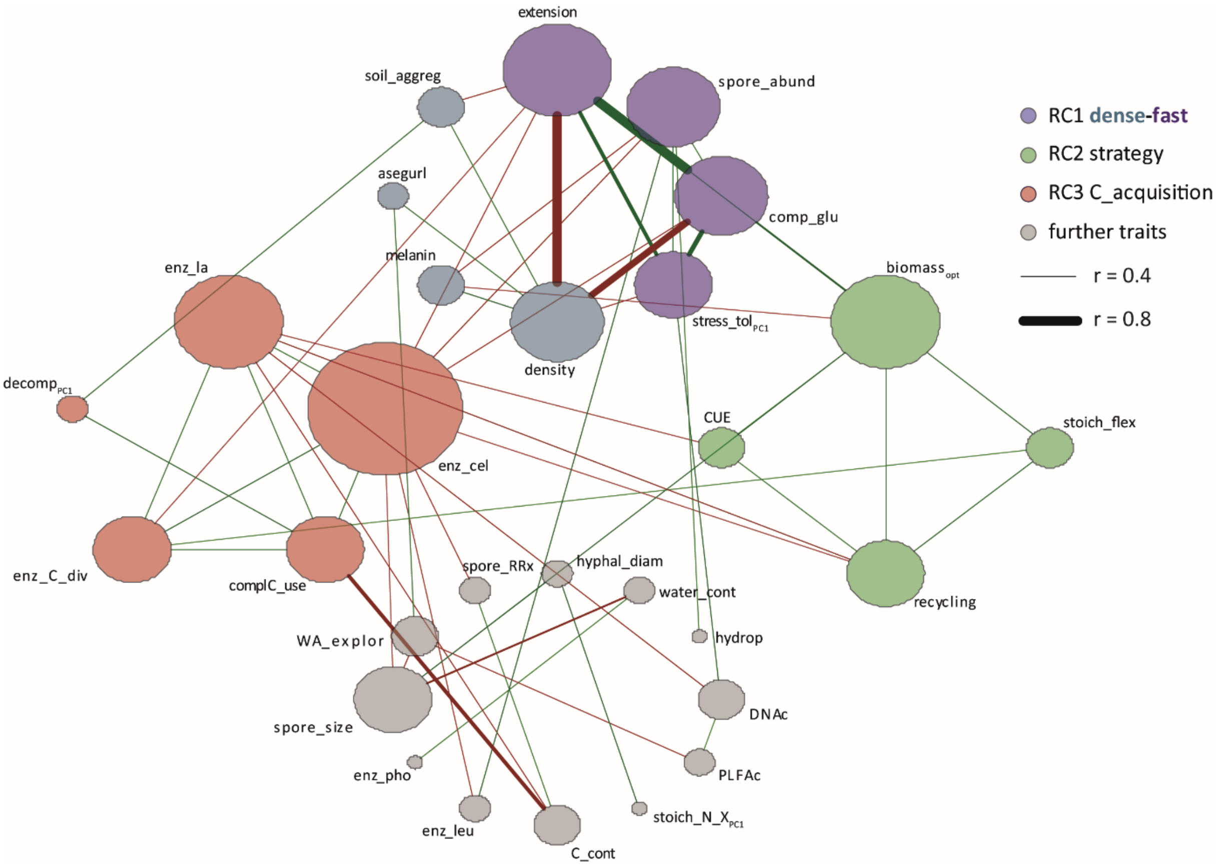

Fig. S1 Pearson correlation among traits visualized as a network. Colours reflect the position of traits on axes of the main PCA (Fig. 3), grey dots represent further fungal traits not included in the main economics space (Fig. 1). Dot sizes reflect the number of connections for each trait. Only significant correlations are displayed ($P < 0.05$).

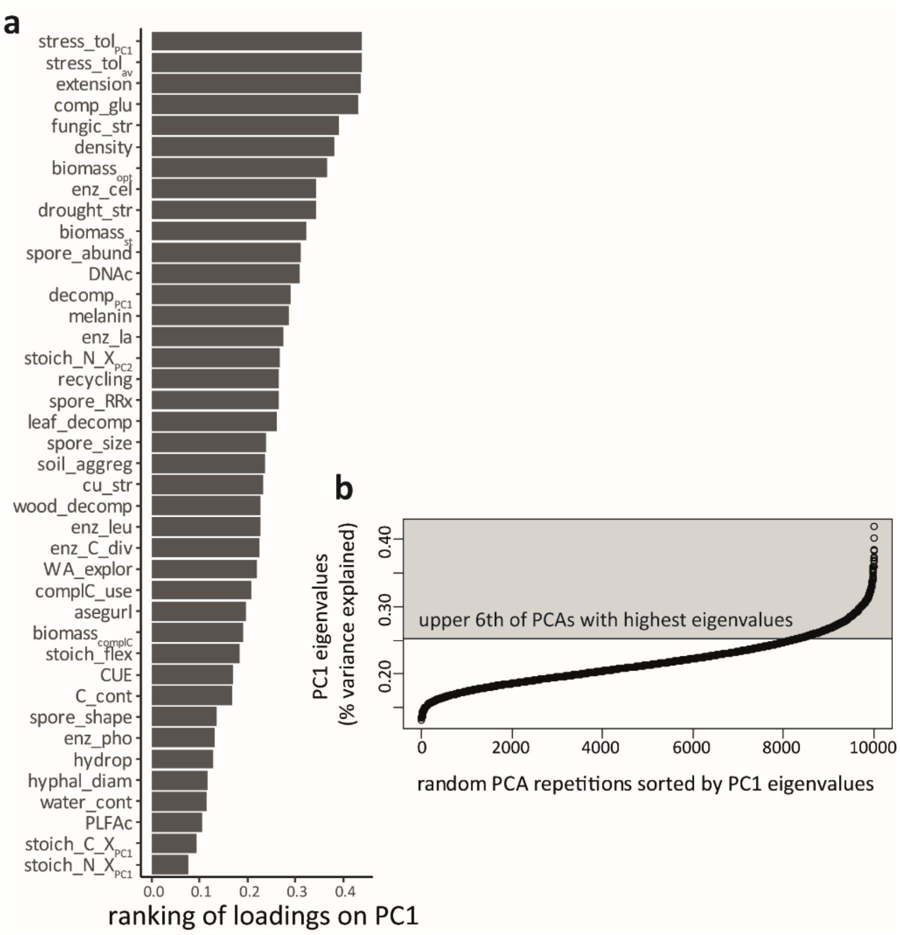

Fig. S2 Relevance of fungal functional traits on the first principal component (PC1) revealed by the PCA randomization method. (**a**) The average absolute loadings of individual traits on PC1 are displayed, based on PCA runs with eigenvalues within the upper sixths of all runs. (**b**) PC1 eigenvalues of all 10,000 random PCA runs are shown, with the upper sixth of PCA runs with highest variance explained marked in grey. 10,000 random PCA repetitions were done, each including 10 randomly selected traits.

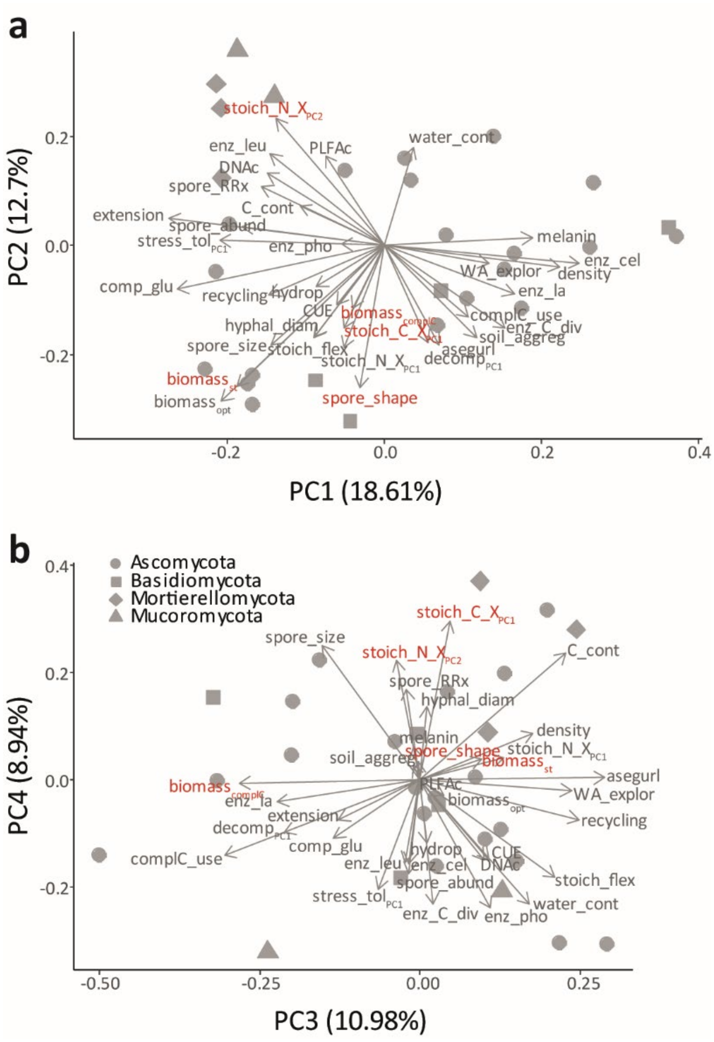

Fig. S3 Principal component analyses (PCA) including all functional traits included in the study. PC1 (principal component 1) and 2 (**a**) and PC 3 and 4 (**b**) are displayed. Arrows represent the loadings of traits on PC axes, dots represent traits of individual isolates with shapes reflecting phylogenetic placements. Traits displayed in red indicate functional traits removed due to co-linearity or low ecological significance.

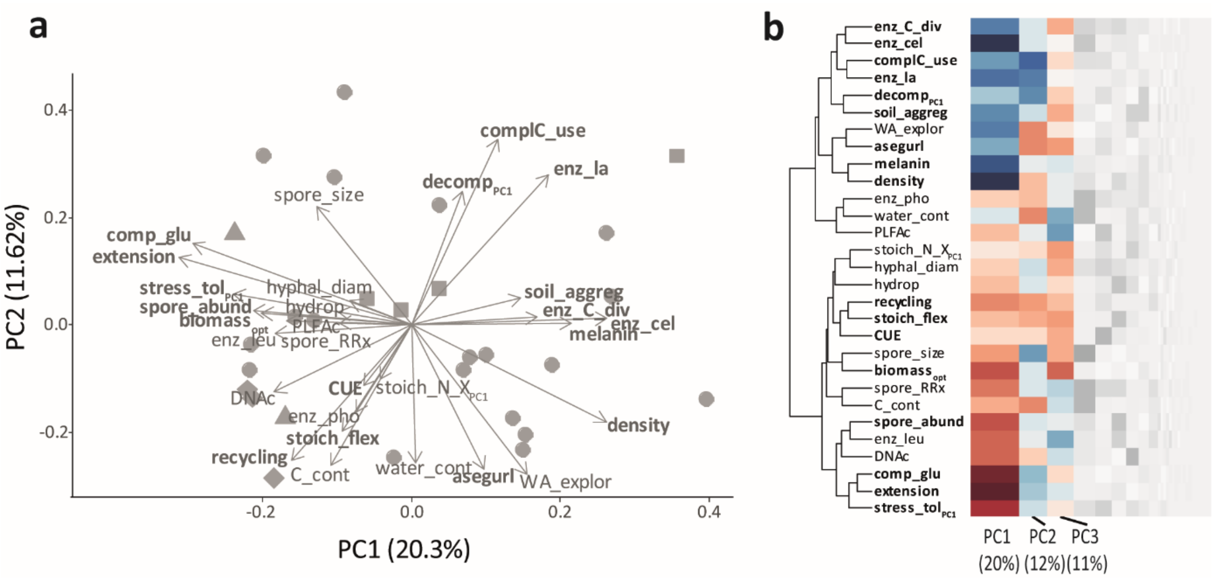

Fig. S4 Visualization of principal component analyses (PCA) including functional traits prior to selection of traits with high functional and ecological significance for saprobic fungal growth in soil. (**a**) PCA results displaying PC axes 1 and 2 (respective eigenvalues in brackets), with arrows showing loadings of traits on PC axes, dots individual isolates (shapes of dots indicate phylogenetic placements). (**b**) Heatmap visualization of the loadings of individual traits on the PC axes, as well as their correlation (displayed as correlogram based on hierarchical cluster analyses). The column width corresponds to the eigenvalues of axes. Only significant axes are displayed in colours (based on PCAtest [23]), where red colours indicate a negative loading, blue colours positive loadings. Colour intensity refers to respective loading strength. Functional traits included in the main economics space (Fig. 3) are displayed in bold.

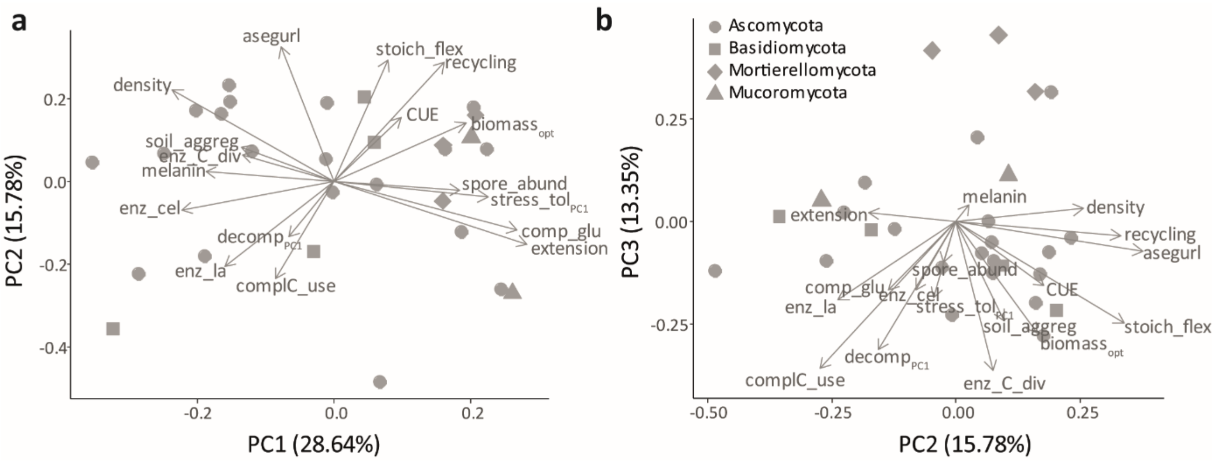

Fig. S5 Visualization of the non-rotated principal component analysis (PCA) of the fungal economics space in saprobic fungal isolates. PC (principal component) axes 1 and PC2 (**a**) and PC2 and PC3 (**b**) are presented. Arrows show eigenvectors of traits on PC axes, dots individual isolates (shapes of dots indicate phylogenetic placement).

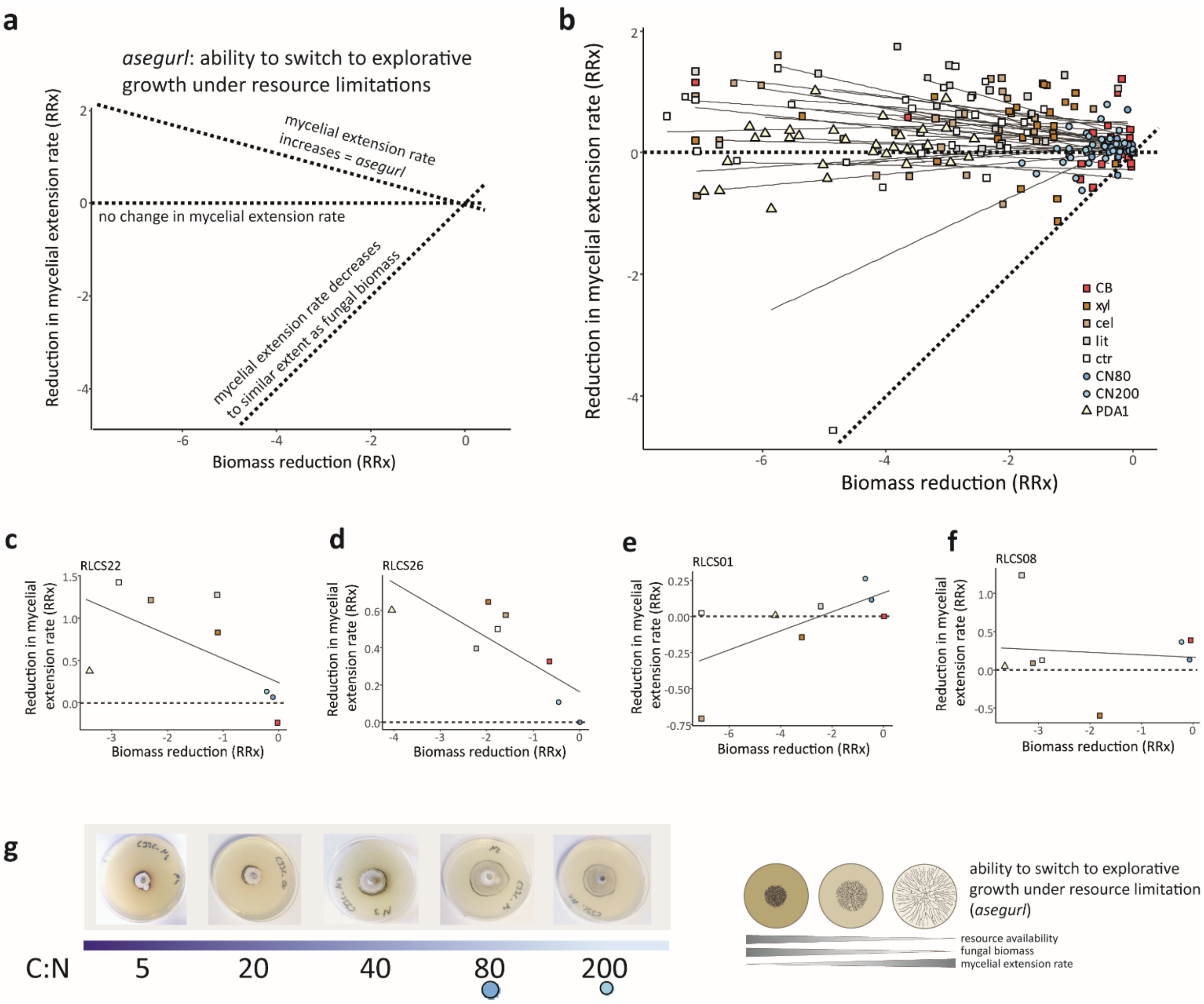

Fig. S6 Overview of the newly defined trait asegurl (ability to switch to explorative growth under resource limitation). (**a - f**) Mycelial extension rate and biomass were measured on different media with reduced resource availability, i.e., more complex C sources (CB: cellobiose, xyl: xylan, cel: cellulose, lit: leaf litter, ctr: no C source added [10]), reduced N contents (defined media with C:N 80 (CN80) or 200 (CN200) [6]) or 1% PDA concentrations (PDA1 [7]) (visualized by dot colours and shapes). The reduction in biomass compared to the isolate-specific maximum biomass at optimum resource supply (natural logarithm (biomass given resource level / maximum biomass at optimum of respective resource gradient) was calculated. Similarly, the simultaneous reduction in mycelial extension rate at this resource level compared to an optimum resource level (based on biomass values) was calculated ((natural logarithm (mycelial extension rate given resource level / mycelial extension rate at optimum (maximum biomass) of respective resource gradient). This reduction in mycelial extension rate was plotted against the reduction in biomass, and the resulting isolate-specific slopes (solid lines) were taken as the isolate-specific values for asegurl. By this, asegurl is higher in case a fungus increases extension rate (explorative growth) despite a growth reduction due to resource limitation. Values above the straight dashed lines (y = 0) show a general increase in mycelial extension, as observed for the majority of samples, while the dashed slope (x = 0 + 1x) indicates the theoretical pattern when extension rate would be affected to the same extent as biomass. (**c - f**) Examples for data of individual isolates. (**g**) Specific example on asegurl observed

along a C:N gradient for an isolate growing on defined media varying in C:N supply (picture modified based on ref [6]; blue dots visualize the two treatments included in the above analyses), related to the visualization of this trait given in Fig. 1. The blue bar indicates the concentration of N available in the medium.

Supplementary Note 2: Niche traits related to environmental parameters

Description of fungal niche traits used in this study can be found in Table S1 and S2.

The fungal growth response to environmental parameters was modeled based on the response curve of biomass values, using a skew-normal distribution with the formula

$$y = a*\exp(k*((x - m))/s - \mathrm{sqrt}(((x - m))/s*((x - m))/s+1)) + b \quad (1)$$

Model fitting was performed using the nonlinear least squares method (nls()) in R version 4.1.3[24]. Based on resulting formulas, the optimum as well as lower and upper niche breadth limits (≥25% of maximum growth) were determined (Fig. 4, S7).

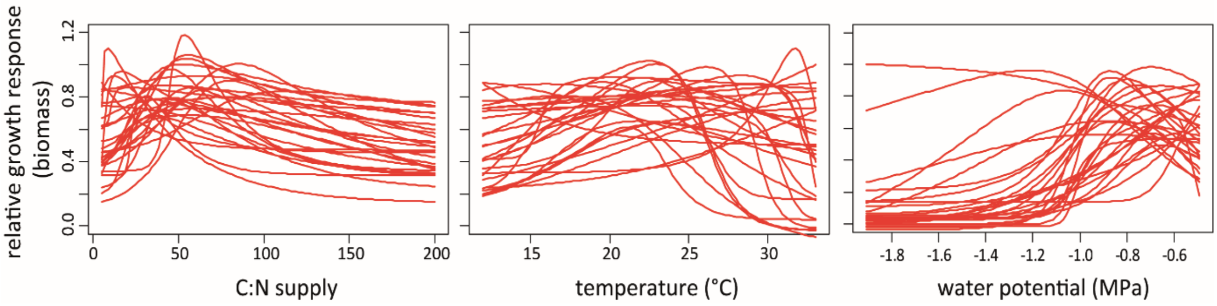

Fig. S7 Relative growth responses of individual fungal isolates modeled along the fundamental niche gradients. Data are based on biomass response curves fitted by skew-normal distributions, relative growth responses for each isolate were standardized by the maximum biomass for each isolate ($\mathrm{biomass}_{rel}[i,j]$ = biomass[i,j] / maximum biomass[i]; i = individual isolate, j = individual data point).

Supplementary Note 3: Methods and results of trait variability analyses

In contrast to plants, the degree of phenotypic plasticity and its influence on trait expressions is not well understood in microorganisms[25, 26], especially for traits that can only be assessed under laboratory conditions. We tested the variability of key fungal traits with varying environmental and substrate conditions in our trait dataset.

In a first experiment, mycelial extension rate, density and biomass, enzymatic activity, fungal C:N ratios and carbon-use efficiency (CUE) of four fungal isolates (RLCS01, RLCS12, RLCS17 and RLCS27; n=3) were compared under varying growth conditions (Fig. S8). Isolates were grown on agar media differing in C:N contents[6], under different temperature regimes as well as on media varying in C substrates. The trait space of individual isolates was compared to the trait space reflecting different growth conditions (Fig. S8a-c). Data measured on media varying in C:N supply (5, 20, 40, 80 and 200)

were already published by Camenzind *et al.*[6]. The experiments testing the effects of temperature and C substrates followed the same experimental design.

Temperature gradient. The four fungal isolates were grown on 10% PDA overlain with cellophane at 15°, 22° and 30°C, a gradient selected based on responses observed in the temperature niche experiment (study CATx, Table S2). Each replicate (n=3) of the same temperature regime was kept in a separate incubator (incubator = experimental unit). Isolates were grown for 7 days, though in case of RLCS01 only 6 days due to its fast growth, whereas RLCS27 needed to grow 21 days to obtain sufficient biomass for C/N analyses. To assess CUE, petri dishes were kept in air-tight sterile 500 ml polypropylene containers [18] and CO_2 was sampled during the whole growth period to avoid O_2 limitations. At the end of growth, small circle segments of the mycelium were cut to determine enzymatic activity following the protocol described by Camenzind *et al.*[6]. Mycelial extension rate was determined by measuring mycelial area with the image-analysis program ImageJ[27], and relating it to the growth period [cm^2 day^{-1}]. The remaining mycelium was transferred to Eppendorf tubes and freeze-dried to determine fungal biomass [mg] and density [mg cm^{-2}]. C and nitrogen (N) contents were analyzed with an Elemental Analyser (EuroEA, HekaTech, Germany). CUE was determined based on the formula.

CUE = (respired C / (respired C + biomass C)) (2)

C substrate gradient. The four fungal isolates were also grown on defined media with different C sources – glucose, cellobiose and xylan. Media were based on phytagel with defined element supply sufficient for fungal growth, and overlain with 1 µm mesh (details described in ref.[10]). Again, fungi were grown for 7 days, except for RLCS01 (6 days) and RLCS 27 (10 days), in closed 500 ml containers. Traits were assessed as described above.

To analyze trait variability explained by isolate identity vs. growth conditions, we plotted the complete trait space of four isolates by principal component analyses (prcomp()). The variance explained by each factor (treatments were modeled as categorical factors) was determined by permutational Multivariate Analysis of Variance (adonis2(), vegan[28]; Fig. 8a-c).

In a <u>second experiment</u>, data on mycelial extension rate, density and fungal biomass assessed for all isolates under varying conditions in C:N, temperature and water availability (see details on niche traits in Table S1 and S2) were analyzed to distinguish the effects of growth condition, isolate identity and phylogeny on the expression of these relevant traits (Fig. S8).

The effects of isolate identity, phylogenetic relatedness and growth condition on individual traits for all 28 isolates was determined by linear regression models for each growth condition, respectively (Fig. S8d). To include phylogeny as an explanatory variable, we applied an eigenvector filtering approach [29]: Following Principal Coordinates Analyses (PCoA, cmdscale()) of the phylogenetic distance matrix (cophenetic.phylo(), ape [30]), axes explaining in sum >80% of variability were extracted. The relative percentage explained by each explanatory variable is based on the sums of squares of resulting linear models. In case of non-linearity, response variables were log-transformed.

<u>As a result</u>, we found that the values of several relevant functional traits tested in four isolates were clearly affected by environmental drivers (Fig. S8a-c). Still, despite clear treatment effects, trait spaces of individual isolates remained separate and unique, with isolate identity explaining higher variances in trait variability than growth condition. Similarly, when analyzing variability in mycelial biomass, density and extension rate in response to different growth conditions for all fungal isolates,

the variability explained by phylogeny and isolate identity by far exceeded the variability explained by treatment (Fig. S8d). This pattern was especially pronounced in mycelial extension rate: C complexity strongly affected fungal biomass and density, but not extension rates (Fig. S8d).

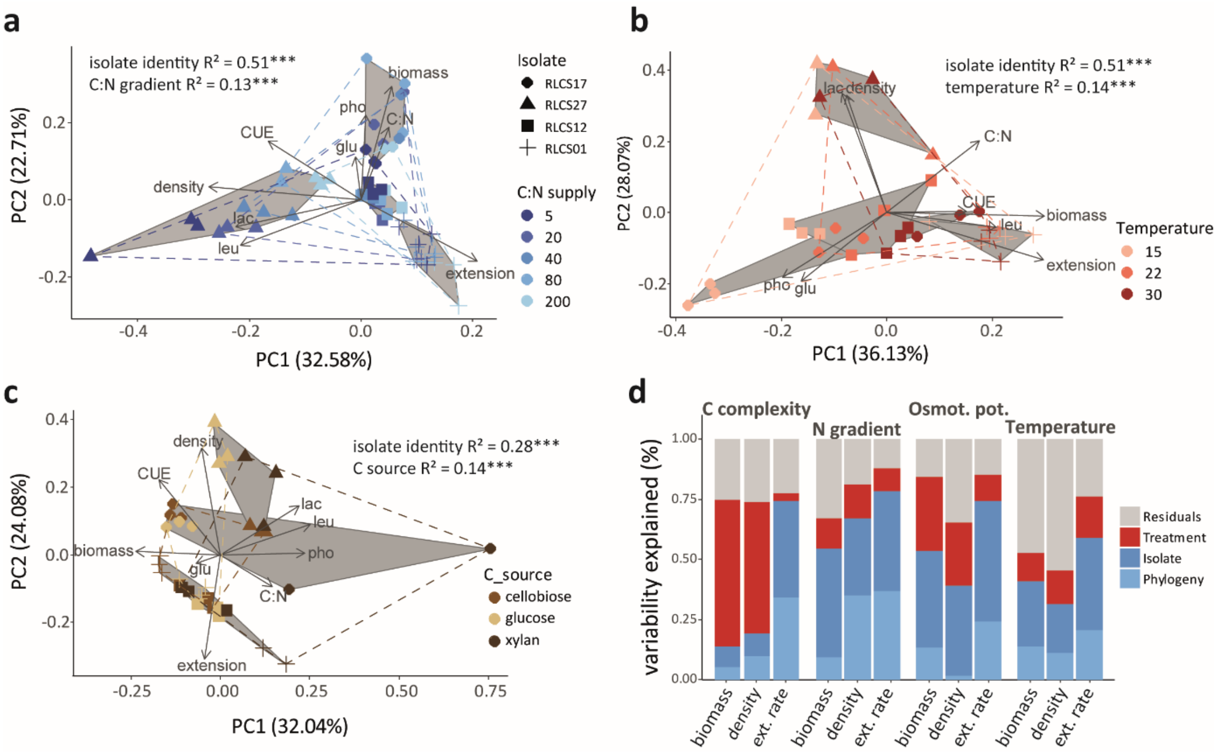

Fig. S8 Variability in main functional traits along varying growth conditions. (**a – c**) Principal component analyses (PCA) of mycelial extension rate (extension), density and biomass, enzymatic activity (lac: laccase, glu: beta-glucosidase, pho: acid phosphatase, leu: leucine aminopeptidase), fungal C:N ratios and carbon-use efficiency (CUE) of four fungal isolates in response to a C:N supply gradient (**a**), varying temperatures (**b**) and different C sources in growth media (**c**). Arrows represent the loadings of traits on the first 2 PC axes, dots represent individual samples, with colours reflecting different growth conditions, and shapes the isolate identity. Grey areas visualize the isolate trait space, dotted lines the environmental trait space. R^2 values and significances (*** $P < 0.001$) based on perMANOVA results are displayed. (**d**) The relative percentage of variability in relevant functional traits (fungal biomass, mycelial density and extension rate) explained by phylogeny, isolate identity or respective treatments is represented by stacked bars. The three trait variables were analyzed in all 28 isolates along each environmental gradient.

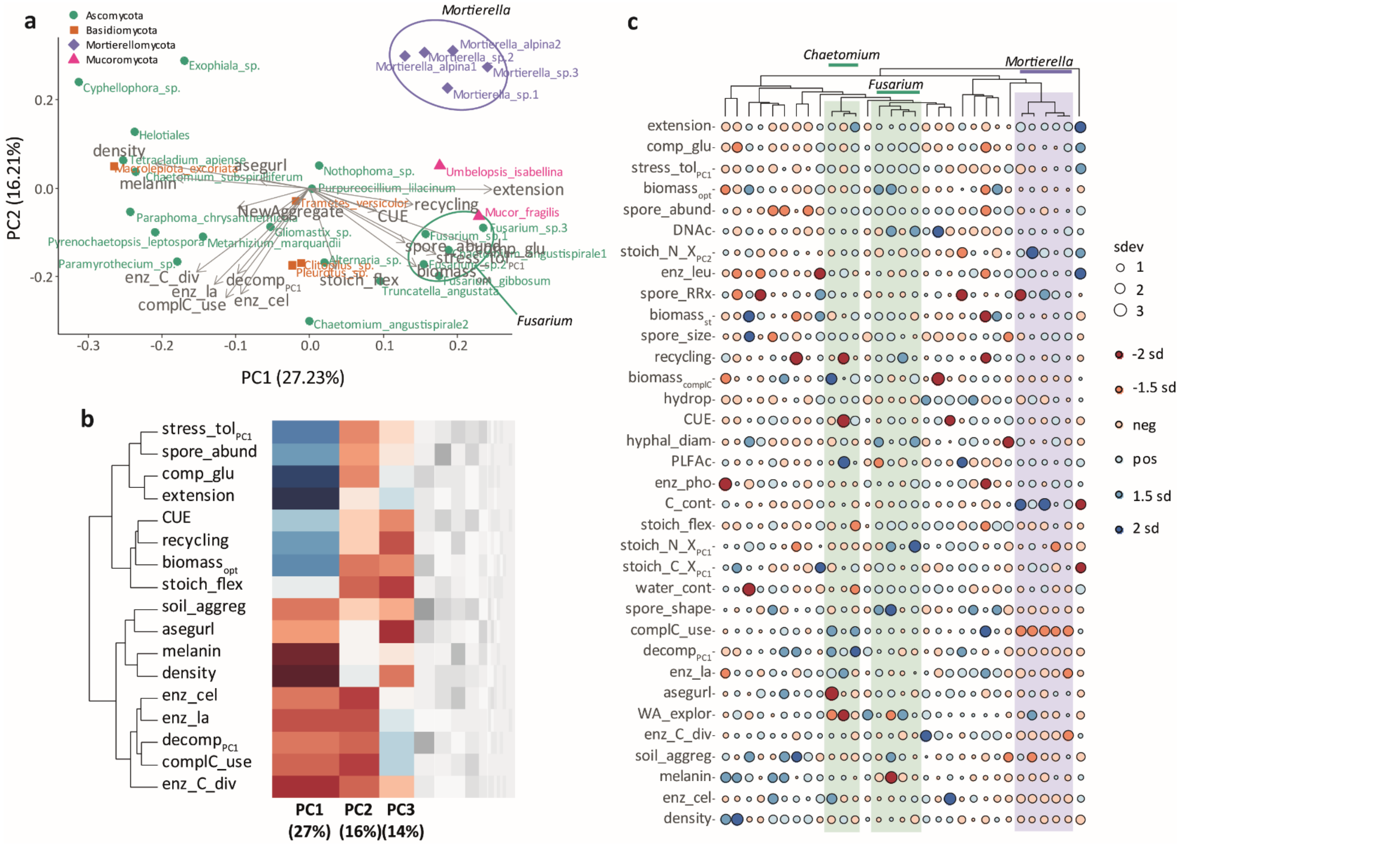

Fig. S9 Trait expressions and correlations in all 31 fungal isolates, including all five *Mortierella* and four *Fusarium* isolates (both reduced to three in the main dataset). When including all isolates, enzymatic traits (C acquisition) group strongly on the first two axes, a pattern driven by the fast-growing *Mortierella* isolates which primarily use simple sugars. (**a**) Visualization of the principal component analysis (PCA) including all isolates – see Fig. 3 and S5 for comparison.

Arrows represent the loadings of traits on the PC axes. Dots the respective position of individual isolates, with colour and dot shapes reflecting respective phyla (see legend). Isolates belonging to the genus *Mortierella* and *Fusarium* are marked by circles that show their close proximity in the trait space. (**b**) Heatmap visualization of the loadings of individual traits on the PC axes, as well as their correlation (displayed as a correlogram based on hierarchical cluster analyses). The column width corresponds to the eigenvalues of axes; asterisks indicate significant loadings of traits on respective axes (based on PCAtest [23]). Red colours indicate a negative loading, blue colours positive loadings. Colour intensity refers to respective loading strength. (**c**) Visualization of standardized trait values for each isolate, sorted by phylogenetic affiliation. Red values indicate negative values, blue colours positive values, dot size reflects the values (based on standard deviations (sd)). Colours indicate isolates with the same genus affiliation. Especially the *Mortierella* isolates have strong similarities in trait expressions.

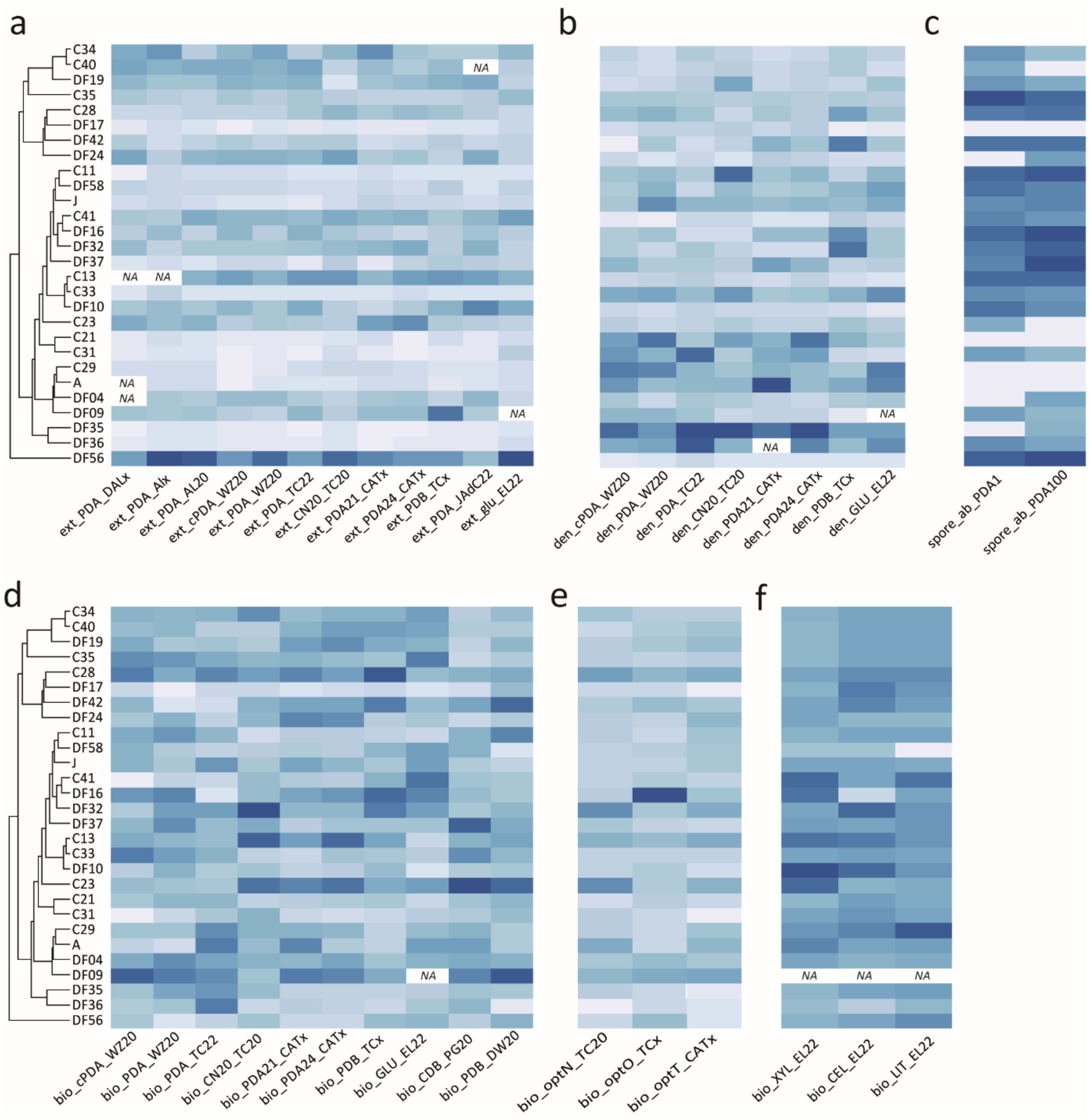

Fig. S10 Heatmaps of individual traits assessed repeatedly under standard conditions used to calculate average values of some main traits, i.e., (**a**) mycelial extension rate, (**b**) mycelial density, (**c**) spore abundance, (**d**) biomass under standard growth conditions, (**e**) biomass under optimal growth conditions and (**f**) biomass on media with complex C. For explanations of trait abbreviations see Table S1. Rows are ordered following the fungal isolate phylogenetic relationship. Blue colours indicate the trait intensity within each individual trait (column), with dark blue indicating high values. Especially mycelial extension rate is highly conserved within isolates, while density and biomass more strongly depend on the medium used (Fig. S8d). All growth media used reflect standard non-stressed conditions.

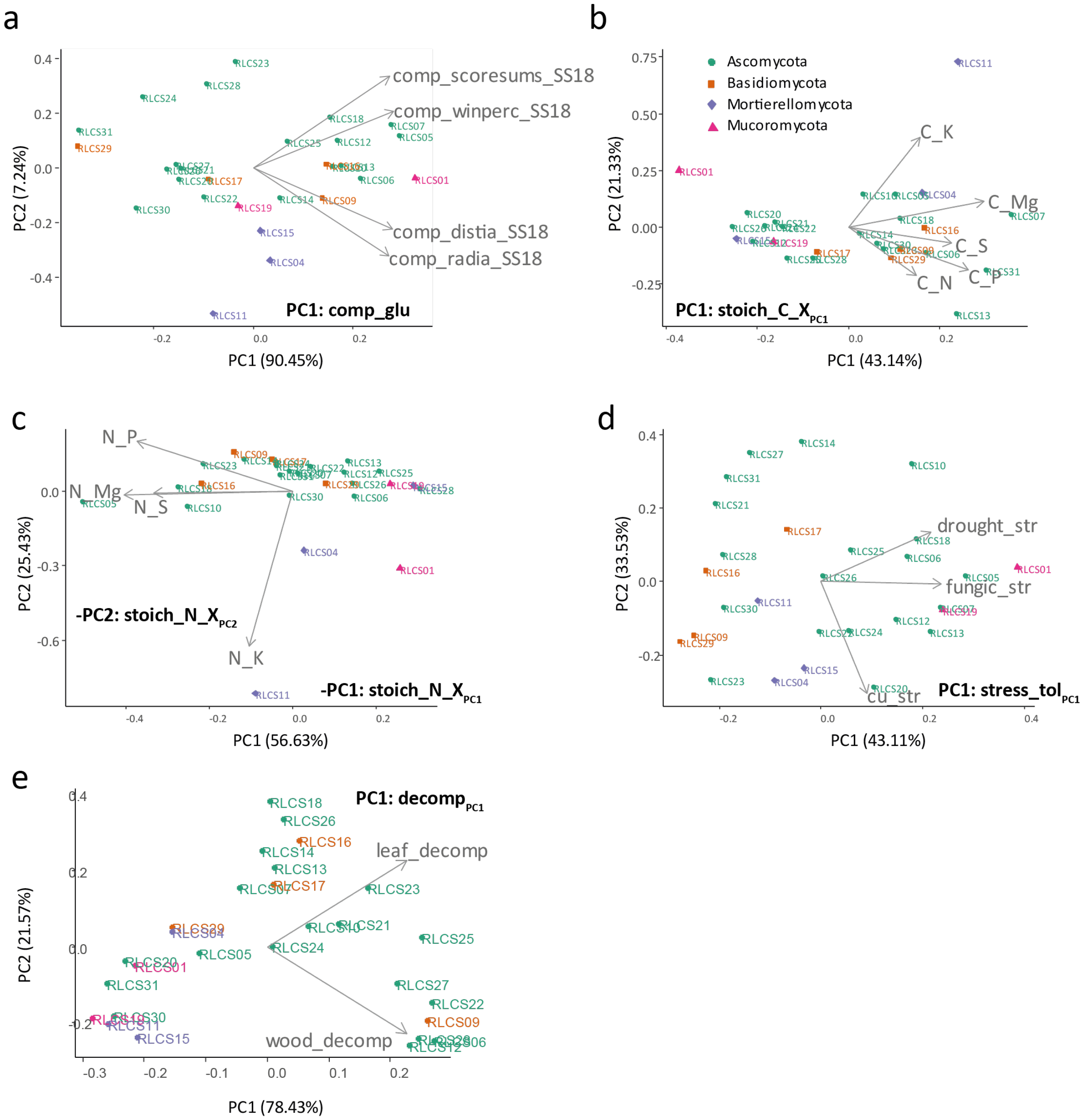

Fig. S11 Extraction of relevant principal component (PC) axes as traits representative of relevant trait syndromes. (**a**) Competitiveness under glucose availability, (**b**) mycelial C:X (nutrient) values, (**c**) mycelial N:X values, (**d**) fungal stress tolerance and (**e**) litter decomposition ability. For explanations of trait abbreviations see Table S1. Dot shape and colour reflect the phylum identity of respective isolates. (**b**,**c**) C:X values are usually strongly driven and affected by C concentrations [8], while N:X values more accurately reflect the element composition of fungi.

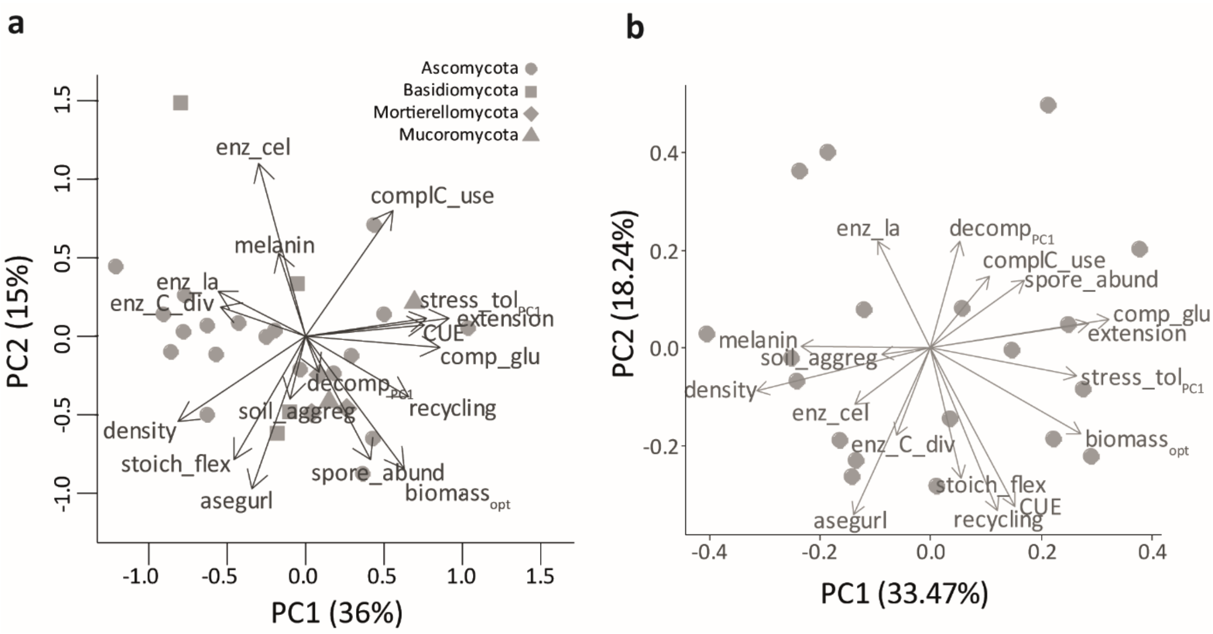

Fig. S12 Visualization of the main fungal economics space in saprobic fungal isolates, based on phylogenetic PCA (phyl.pca(), phytools[31]), implementing a Brownian motion model (**a**) and Ascomycota only (**b**). Principal component analysis (PCA) of functional traits display PC axes 1 and 2 (respective eigenvalues in brackets), with arrows showing loadings of traits on PC axes, dots individual isolates (with shapes reflecting phylogenetic placement).

References

1. Fernandez CW, Koide RT. Initial melanin and nitrogen concentrations control the decomposition of ectomycorrhizal fungal litter. *Soil Biology and Biochemistry* **77**, 150-157 (2014).

2. Lehmann A, Zheng W, Soutschek K, Roy J, Yurkov AM, Rillig MC. Tradeoffs in hyphal traits determine mycelium architecture in saprobic fungi. *Scientific Reports* **9**, 14152 (2019).

3. Lehmann A, *et al.* Fungal Traits Important for Soil Aggregation. *Frontiers in Microbiology* **10**, 2904 (2020).

4. Zheng W, Lehmann A, Ryo M, Vályi KK, Rillig MC. Growth rate trades off with enzymatic investment in soil filamentous fungi*. Scientific Reports* **10**, 11013 (2020).

5. Zheng W. Soil stability and filamentous fungi. Doctoral thesis. Institute of Biology, Freie Universität Berlin, http://dx.doi.org/10.17169/refubium-8339 (2015).

6. Camenzind T, Lehmann A, Ahland J, Rumpel S, Rillig M. Trait-based approaches reveal fungal adaptations to nutrient-limiting conditions. *Environmental Microbiology* **22**, 3548-3560 (2020).

7. Camenzind T, Weimershaus P, Lehmann A, Aguilar-Trigueros C, Rillig MC. Soil fungi invest into asexual sporulation under resource scarcity, but trait spaces of individual isolates are unique. *Environmental Microbiology* **24**, 2962-2978 (2022).

8. Camenzind T, Philipp Grenz K, Lehmann J, Rillig MC. Soil fungal mycelia have unexpectedly flexible stoichiometric C:N and C:P ratios. *Ecology Letters* **24**, 208-218 (2021).

9. dela Cruz JA, Camenzind T, Rillig MC. Sub-lethal fungicide concentrations both reduce and stimulate the growth rate of non-target soil fungi from a natural grassland. *Frontiers in Environmental Science* **10**, 1020465 (2022).

10. Leifheit E, *et al.* Fungal traits help to understand the decomposition of simple and complex plant litter. *Fems Microbiology Ecology*, in press, doi: 10.1093/femsec/fiae033 (2024).

11. Golubeva P, *et al.* Soil Saprobic Fungi Differ in Their Response to Gradually and Abruptly Delivered Copper. *Front Microbiol* **11**, 1195 (2020).

12. Wang D. Trade-offs in soil filamentous fungi. Doctoral thesis. Institute of Biology, Freie Universität Berlin, http://dx.doi.org/10.17169/refubium-27326 (2020).

13. Soliveres S, *et al.* Intransitive competition is common across five major taxonomic groups and is driven by productivity, competitive rank and functional traits. *Journal of Ecology* **106**, 852-864 (2018).

14. Veresoglou SD, Wang D, Andrade-Linares DR, Hempel S, Rillig MC. Fungal Decision to Exploit or Explore Depends on Growth Rate. *Microbial Ecology* **75**, 289-292 (2018).

15. Aujla IS, Paulitz TC. An Improved Method for Establishing Accurate Water Potential Levels at Different Temperatures in Growth Media. *Front Microbiol* **8**, 1497 (2017).

16. Palacios S, Casasnovas F, Ramirez ML, Reynoso MM, Torres AM. Impact of water potential on growth and germination of Fusarium solani soilborne pathogen of peanut. *Brazilian journal of microbiology* **45**, 1105-1112 (2014).

17. Frostegård A, Tunlid A, Baath E. Phospholipid fatty-acid composition, biomass and activity of microbial communities from two soil types experimentally exposed to different heavy-metals. *Applied and Environmental Microbiology* **59**, 3605-3617 (1993).

18. Maynard DS, Crowther TW, Bradford MA. Fungal interactions reduce carbon use efficiency. *Ecology Letters* **20**, 1034-1042 (2017).

19. Bills GF, Foster MS. Formulae for Selected Materials Used to Isolate and Study Fungi and Fungal Allies. In: Biodiversity of Fungi (eds Mueller GM, Bills GF, Foster MS). Academic Press (2004).

20. Nilsson RH, *et al.* The UNITE database for molecular identification of fungi: handling dark taxa and parallel taxonomic classifications. *Nucleic Acids Research* **47**, D259-D264 (2018).

21. Cole JR, *et al.* Ribosomal Database Project: data and tools for high throughput rRNA analysis. *Nucleic acids research* **42**, D633-D642 (2014).

22. Callahan BJ, McMurdie PJ, Rosen MJ, Han AW, Johnson AJA, Holmes SP. DADA2: High-resolution sample inference from Illumina amplicon data. *Nature Methods* **13**, 581-583 (2016).

23. Camargo A. PCAtest: testing the statistical significance of Principal Component Analysis in R. *PeerJ* **10**, e12967 (2022).

24. R Core Team. R: A language and environment for statistical computing.). R Foundation for Statistical Computing (2021).

25. Nicotra AB, *et al.* Plant phenotypic plasticity in a changing climate. *Trends in plant science* **15**, 684-692 (2010).

26. Alster CJ, Allison SD, Johnson NG, Glassman SI, Treseder KK. Phenotypic plasticity of fungal traits in response to moisture and temperature. *ISME Communications* **1**, 43 (2021).

27. Schneider CA, Rasband WS, Eliceiri KW. NIH Image to ImageJ: 25 years of image analysis. *Nature Methods* **9**, 671-675 (2012).

28. Oksanen J, *et al.* vegan: Community Ecology Package. R package version 2.6-2. edn (2022).

29. Hempel S, *et al.* Mycorrhizas in the Central European flora: relationships with plant life history traits and ecology. *Ecology* **94**, 1389-1399 (2013).

30. Paradis E, Schliep K. ape 5.0: an environment for modern phylogenetics and evolutionary analyses in R. *Bioinformatics* **35**, 526-528 (2018).

31. Revell LJ. phytools: an R package for phylogenetic comparative biology (and other things). *Methods in Ecology and Evolution* **3**, 217-223 (2012).

32. Camenzind T, Haslwimmer H, Rillig MC, Ruess L, Finn DR, Tebbe CC, Hempel S, Marhan S. Revisiting soil fungal biomarkers and conversion factors: Interspecific variability in phospholipid fatty acids, ergosterol and rDNA copy numbers. *Soil Ecology Letters*, in press, (2024)
