# Supplementary material for: Towards establishing a fungal economics spectrum in soil saprobic fungi
Source: Nat Commun. 2024 Apr 18;15:3321. doi: 10.1038/s41467-024-47705-7 (PMC11026409; doi:10.1038/s41467-024-47705-7)
Supplement: Supplementary file 3 — Reporting Summary [file 41467_2024_47705_MOESM3_ESM.pdf]

nature portfolio

Corresponding author(s): Tessa Camenzind

Last updated by author(s): Mar 30, 2024

Reporting Summary

Nature Portfolio wishes to improve the reproducibility of the work that we publish. This form provides structure for consistency and transparency in reporting. For further information on Nature Portfolio policies, see our Editorial Policies and the Editorial Policy Checklist.

Statistics

For all statistical analyses, confirm that the following items are present in the figure legend, table legend, main text, or Methods section.

n/a	Confirmed	
☐	☒	The exact sample size (*n*) for each experimental group/condition, given as a discrete number and unit of measurement
☐	☒	A statement on whether measurements were taken from distinct samples or whether the same sample was measured repeatedly
☐	☒	The statistical test(s) used AND whether they are one- or two-sided<br>*Only common tests should be described solely by name; describe more complex techniques in the Methods section.*
☐	☒	A description of all covariates tested
☐	☒	A description of any assumptions or corrections, such as tests of normality and adjustment for multiple comparisons
☐	☒	A full description of the statistical parameters including central tendency (e.g. means) or other basic estimates (e.g. regression coefficient) AND variation (e.g. standard deviation) or associated estimates of uncertainty (e.g. confidence intervals)
☐	☒	For null hypothesis testing, the test statistic (e.g. *F*, *t*, *r*) with confidence intervals, effect sizes, degrees of freedom and *P* value noted<br>*Give P values as exact values whenever suitable.*
☒	☐	For Bayesian analysis, information on the choice of priors and Markov chain Monte Carlo settings
☒	☐	For hierarchical and complex designs, identification of the appropriate level for tests and full reporting of outcomes
☐	☒	Estimates of effect sizes (e.g. Cohen's *d*, Pearson's *r*), indicating how they were calculated

Our web collection on statistics for biologists contains articles on many of the points above.

Software and code

Policy information about availability of computer code

Data collection	No software was used for data collection.
Data analysis	Statistical analyses were conducted with open source R version 4.1.3. R packages used for statistical analyses include DECIPHER (2.22.0), phangorn (2.8.1), PCAtest (0.0.1), phytools (1.0-1), vegan (2.6-2). Visual illustrations are supported by packages ggplot2 (3.3.5), ggfortify (0.4.14), RColorBrewer (1.1-2), pca3d (0.10.2), qgraph (1.9.2), Hmisc (4.7-1), forcats (0.5.1), scatterplot3d (0.3-41), plot3D (1.4), corrplot (0.92), tidyr (1.2.0). Phylogenetic analyses base on R packages ape (5.6-2), phytools (1.0-1), phylosignal (1.3), phylobase (0.8.10).<br>All statistical code is available as open resource at figshare, https://doi.org/10.6084/m9.figshare.23320148.

For manuscripts utilizing custom algorithms or software that are central to the research but not yet described in published literature, software must be made available to editors and reviewers. We strongly encourage code deposition in a community repository (e.g. GitHub). See the Nature Portfolio guidelines for submitting code & software for further information.

Data

Policy information about availability of data

All manuscripts must include a data availability statement. This statement should provide the following information, where applicable:

- Accession codes, unique identifiers, or web links for publicly available datasets
- A description of any restrictions on data availability
- For clinical datasets or third party data, please ensure that the statement adheres to our policy

All fungal functional trait data analyzed in this study have been deposited in figshare under the DOI https://doi.org/10.6084/m9.figshare.23320148. This data is publicly available.
Cultures of fungal isolates and respective sequences were deposited at NCBI (National Center for Biotechnology) and the DSMZ (German Collection of Microorganisms and Cell Cultures GmbH). Accession numbers are available in Supplementary Information Table S3.

Research involving human participants, their data, or biological material

Policy information about studies with human participants or human data. See also policy information about sex, gender (identity/presentation), and sexual orientation and race, ethnicity and racism.

Reporting on sex and gender	N/A
Reporting on race, ethnicity, or other socially relevant groupings	N/A
Population characteristics	N/A
Recruitment	N/A
Ethics oversight	N/A

Note that full information on the approval of the study protocol must also be provided in the manuscript.

Field-specific reporting

Please select the one below that is the best fit for your research. If you are not sure, read the appropriate sections before making your selection.

☐ Life sciences ☐ Behavioural & social sciences ☒ Ecological, evolutionary & environmental sciences

For a reference copy of the document with all sections, see nature.com/documents/nr-reporting-summary-flat.pdf

Ecological, evolutionary & environmental sciences study design

All studies must disclose on these points even when the disclosure is negative.

Study description	In this study we analyzed a collection of fungal trait data in a set of 28 saprobic soil fungi isolated from a natural grassland soil. The correlation and main trade-offs in fungal traits were determined, and compared with fundamental niche gradients of individual isolates.
Research sample	We used saprobic fungal isolates derived from a natural grassland site in Northern Germany. Fungi were isolated in 2014, using techniques to include a diverse phylogenetic set.<br>The identity of the fungi was analyzed by long read sequencing (ITS1, 5.8S, ITS2 and partial LSU). Isolates and respective sequences were deposited at NCBI (National Center for Biotechnology) and the DSMZ (German Collection of Microorganisms and Cell Cultures GmbH).<br>A list of these fungal isolates and information on their identity and accession numbers is provided in the Supporting Information Table S3.
Sampling strategy	From the fungal strains obtained by soil isolation, an original collection of 31 fungal isolates were kept in the Rillig Lab Coreset for further trait analyses. These 31 isolates were selected to maximize high phylogenetic coverage. In this study, three of these fungal isolates were further excluded to reduce the phylogenetic signal. The number of isolates included here (28) is exceeding or equal to previous research on fungal trait studies. It was not possible to include more isolates, since many trait measurement are highly laborious. In this study we aimed to include all ecologically relevant functional traits to define a fungal economics spectrum, an approach that did not allow to include more isolates than presented here.
Data collection	Fungal trait data were assessed by authors of this manuscript since 2014. Fungal traits were determined in separate experiments, though the experimental setup was overall comparable (concerning conditions and duration of experiments). Detailed information about indvidual studies and traits included are listed in the Supporting Information.

Timing and spatial scale	Individual fungal isolates were isolated from soil samples in 2013/2014. Since then these isolates were kept on potato-dextrose agar at 4°C, and refreshed regularly from stock cultures. Separate fungal trait experiments were conducted between 2014 and 2022.
Data exclusions	Data from three fungal isolates were excluded (originally this fungal collection consisted of 31 fungal isolates), in order to reduce the phylogenetic bias in the data.
Reproducibility	Since this study represents an analysis of one large trait collection, reproducibility does not apply at this stage; there is no comparable fungal trait collection to repeat these analyses. However, in a follow-up experiment with other fungal isolates we found similar principal component axes as defined here, based on few key fungal traits tested (unpublished data).<br>Certain limitations, e.g., due to trait variability, were reduced by combining repeated measurements of individual traits, or extracting trait syndromes from similar fungal traits fused by extraction of principal component axes.
Randomization	All individual trait studies applied standard randomization approaches in fungal growth designs, where applicable.
Blinding	Blinding was applied in trait measurements when testing for treatment effects.

Did the study involve field work? ☒ Yes ☐ No

Field work, collection and transport

Field conditions	During the sampling times this region has an average temperature of 15°C and ~50mm of rainfall per month (see for example https://en.climate-data.org/europe/germany/brandenburg-424/)
Location	Fungal strains were isolated from soil samples taken in a natural grassland area of Northeastern Germany ('Oderhänge Mallnow' close to the town of Lebus, Germany; 52°28`N, 14°29`E)
Access & import/export	A sampling permit (RO7/SOB-0951A to I) was obtained from the federal environmental agency Brandenburg (Landesumweltamt Brandenburg, Außenstelle Frankfurt/Oder, RO 7 - Naturschutz).
Disturbance	There was low disturbance, only few soil samples were taken.

Reporting for specific materials, systems and methods

We require information from authors about some types of materials, experimental systems and methods used in many studies. Here, indicate whether each material, system or method listed is relevant to your study. If you are not sure if a list item applies to your research, read the appropriate section before selecting a response.

Materials & experimental systems

n/a	Involved in the study
☒	☐ Antibodies
☒	☐ Eukaryotic cell lines
☒	☐ Palaeontology and archaeology
☐	☒ Animals and other organisms
☒	☐ Clinical data
☒	☐ Dual use research of concern
☒	☐ Plants

Methods

n/a	Involved in the study
☒	☐ ChIP-seq
☒	☐ Flow cytometry
☒	☐ MRI-based neuroimaging

Animals and other research organisms

Policy information about studies involving animals; ARRIVE guidelines recommended for reporting animal research, and Sex and Gender in Research

Laboratory animals	This study did not involve laboratory animals. Saprobic fungal isolates obtained from natural grassland soil were analyzed in this study.
Wild animals	N/A
Reporting on sex	N/A
Field-collected samples	Fungal isolates were obtained from soil samples, and maintained on agar plates or as stock cultures (conserved either in 10% glycerol (-80°C), mineral oil (4°C) or 20% skim milk (freeze dried, 4°C)).
Ethics oversight	No ethical approval is required to work with soil fungi.

Note that full information on the approval of the study protocol must also be provided in the manuscript.

Plants

Seed stocks	N/A
Novel plant genotypes	N/A
Authentication	N/A
